# Supplementary material for: The interactome of intact mitochondria by cross-linking mass spectrometry provides evidence for coexisting respiratory supercomplexes
Source: Mol Cell Proteomics. 2017 Dec 8;17(2):216–32. doi: 10.1074/mcp.RA117.000470 (PMC5795388; doi:10.1074/mcp.RA117.000470)
Supplement: Supplemental Data [file supp_RA117.000470_133922_0_supp_23978_fzffwf.zip › spectra_annotation/mito_spectra_annotation/283-1-3-1-4-1.pdf]

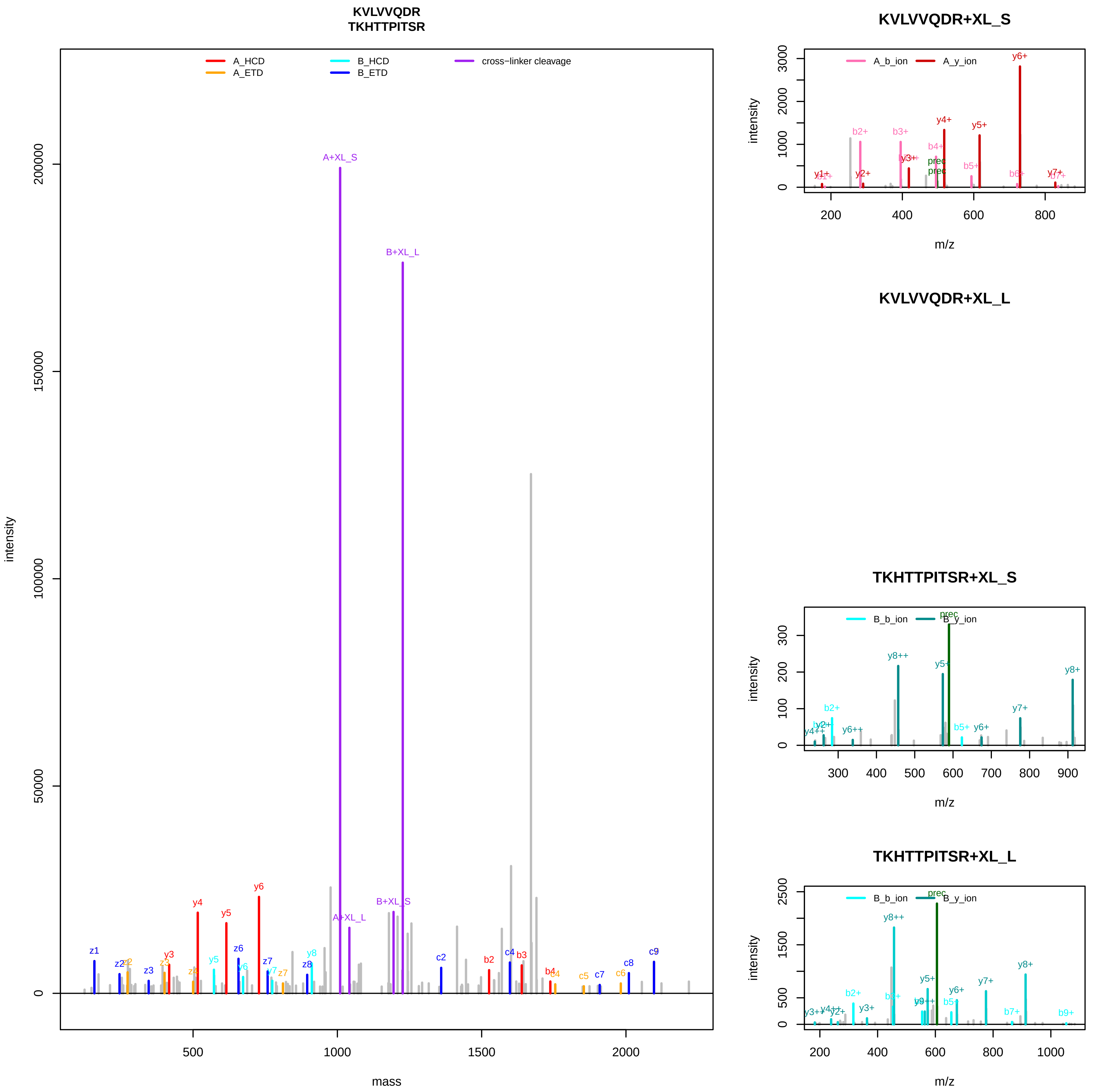

KVLVVQDR
TKHTTPITSR
A_HCD
A_ETD
B_HCD
B_ETD
cross−linker cleavage
A+XL_S
B+XL_L
B+XL_S
A+XL_L
intensity
mass
0
50000
100000
150000
200000
500
1000
1500
2000
z1
z2
z3
z6
z7
z8
y3
y4
y5
y6
y7
y8
c2
c4
c5
c6
c7
c8
c9
b2
b3
b4
KVLVVQDR+XL_S
A_b_ion
A_y_ion
y1+
y2+
y3+
y4+
y5+
y6+
y7+
b2+
b3+
b4+
b5+
b6+
prec
m/z
KVLVVQDR+XL_L
TKHTTPITSR+XL_S
B_b_ion
B_y_ion
y4++
y6++
y8++
y5+
y6+
y7+
y8+
b2+
b5+
TKHTTPITSR+XL_L
y3++
y4++
y2+
y3+
y9++
b7+
b9+
